# 12-month outcomes of ab interno excisional goniotomy combined with cataract surgery in primary open-angle glaucoma and normal tension glaucoma

**DOI:** 10.1007/s10792-023-02659-5

**Published:** 2023-03-02

**Authors:** David Kuerten, Peter Walter, Sabine Baumgarten, Matthias Fuest, Niklas Plange

**Affiliations:** Department of Ophthalmology, RWTH Aachen University, Pauwelsstr. 30, 52057 Aachen, Germany

**Keywords:** Normal tension glaucoma, Kahook Dual Blade, MIGS, Phacoemulsification

## Abstract

**Purpose:**

To evaluate the efficacy and safety of excisional goniotomy performed with the Kahook Dual Blade (KDB) combined with cataract surgery in patients with pimary open angle glaucoma (POAG) and Normal Tension Glaucoma (NTG) under topical therapy. Further sub-analysis was performed to compare between 90 and 120 degrees goniotomy.

**Methods:**

This was a prospective case series of 69 eyes from 69 adults (age 78 ± 5.9 years; male = 27, female = 42). Indications for surgery included insufficient IOP control with topical medication, glaucomatous damage progression under topical therapy and reduction of medication burden. Complete success was defined as IOP lowering below 21 mmHg without the need for topical medication. For NTG patients, complete success was defined as IOP lowering below 17 mmHg without the need for topical medication.

**Results:**

IOP was significantly lowered from 19.7 ± 4.7 to 15.1 ± 2.7 at 2 months, 15.8 ± 2.3

at 6 months and 16.1 ± 3.2 at 12 months (*p *< 0.05) for POAG and 15.1 ± 2.5 to 14.1 ± 2.4 at 2 months, 14.1 ± 3.1 at 6 months and 13.6 ± 1.8 at 12 months (*p *> 0.08) for NTG, respectively. Complete success was achieved in 64% of the patients. IOP lowering under 17 mmHg without the need for topical medication was achieved in 60% of the patients at 12 months. In NTG patients (14 eyes) IOP lowering under 17 mmHg without the need for topical medication was achieved in 71%. No significant difference was recorded in terms of IOP lowering at 12 months in-between 90° and 120° of treated trabecular meshwork (*p *> 0.7). No severe adverse reactions were recorded in this study.

**Conclusion:**

One-year results show that KDB combined with cataract surgery is an effective treatment option for glaucoma patients. IOP lowering was successfully achieved in NTG patients with complete success in 70% of the patients. In our study, no significant differences were recorded in-between 90° and 120° of treated trabecular meshwork.

## Introduction

Surgical treatment of glaucoma is undergoing an evolution in recent years. The development of minimally invasive glaucoma surgery (MIGS) that offer significant intraocular pressure (IOP) reduction, while having fewer adverse events than more traditional surgical procedures, leads to a steady increase in glaucoma surgeries [1]. MIGS is collectively characterized as minimally invasive techniques mostly using an ab interno approach with minimal disruption of the normal ocular anatomy and is supposed to provide rapid visual recovery [2]. As the enlarging lens with age was identified as a risk factor in open angle and particularly angle closure glaucoma [3] pairing these MIGS procedures with cataract surgery seemed like an obvious approach.

The Kahook Dual Blade (KDB) procedure is an advancement of traditional goniotomy, first described in the 1930s [4]. The success rates of traditional goniotomy have been reported to range from 50 to 90% in different populations [5]. The KDB was designed to manually excise a strip of trabecular meshwork, 230 µm wide, which may allow more filtration than simple incision in traditional goniotomy [6, 7]. After piercing the TM the blade is advanced in a circumferential manner, thereby the TM is stretched over the ramp of the blade and 2 parallel incisions are created. Subsequently, the created strip can be removed allowing natural, unhindered aqueous outflow into the Schlemm’s canal. Usually, 120° of the trabecular meshwork circumference is treated. No filtering bleb is created, and no foreign object is permanently implanted. Promising results in adults as well as juvenile open-angle glaucoma patients have been reported [8–10]. IOP reduction was between 24 and 36% and medication burden was reduced by 37–70% through 6–12 months follow-up in different studies [6, 9, 11, 12].

In this study, we investigated the efficacy and real-life safety of KDB combined with phacoemulsification in patients with primary open-angle glaucoma (POAG) and Normal tension glaucoma (NTG). Further analysis was performed if 90° vs 120° degrees circumferential KDB showed significant differences in terms of IOP/medication burden reduction.

## Methods

This was a prospective interventional case series. All procedures performed in studies involving human participants were in accordance with the ethical standards of the institutional and/or national research committee and with the 1964 Declaration of Helsinki and its later amendments or comparable ethical standards. The study was conducted under a waiver of consent following review of a central ethics board (Nr. 2021294, Ethics committee Aerztekammer Nordrheinwestfalen).

Only adult subjects diagnosed with both visually significant cataract and open-angle glaucoma, that were on topical IOP lowering medication and showed glaucomatous visual field damage and/or glaucomatous optic neuropathy as defined by the European glaucoma society, were included in this study. Only eyes with uneventful cataract surgery (e.g., no vitreous loss, no IOL implantation outside of the capsular back) were included, as this study was aimed at investigating KDBs efficacy and safety. All eyes included in this prospective case series completed the 12-month follow-up. (16 patients were lost to the 12-months follow-up and therefore excluded from the analysis.)

All eyes did not receive other glaucoma surgeries prior to the inclusion and no eyes needed other glaucoma surgeries in the 12 months after combined KDB and cataract surgery. All surgeries were performed by the same experienced surgeon (NP). Each surgery was performed as recommended by the manufacturers, following completion of cataract extraction and IOL placement, KDB was performed for either 90 or 120 degrees. Surgeries with successful KDB between 100 and 110 degrees were not included in the sub-analysis (90 vs. 120 degrees).

Baseline data were collected from a preoperative visit about 4 weeks prior to surgery; postoperative data were collected at 2 months, 6 months and 12 months after surgery. The data included demographics (age at surgery, sex, laterality of glaucoma), glaucoma status, IOP, IOP lowering medication, visual acuity and adverse events at every postoperative visit. IOP was measured using Goldmann applanation tonometry.

Complete surgical success was defined as IOP lowering below 21 mmHg without the need for topical medication. In NTG patients complete success was defined as IOP below 17 mmHg without the need for topical medication.

### KDB excisional goniotomy

The angulation of the microscope was adjusted and the patient’s head was tilted to allow visualization of the nasal chamber angle, using a single-use gonioprism lens (MV LV 48, Phakos, Montreuil, France). A clear cornea temporal incision was created using a 23 G microincision vitrectomy blade for cataract surgery, and a cohesive ophthalmic viscoelastic (Albomed, Germany) was used to deepen the anterior chamber. The KDB was introduced through the incision and advanced across the AC until it reached the nasal angle. Under direct gonioscopic visualization, the tip of KDB was used to create an incision in the TM, placing the footplate against the inner wall of SC, then advancing the blade along the TM, in an anticlockwise (forehand) direction for 90° degrees. In cases of 120° degree KDB the blade was advanced from the primary starting point in a clockwise direction (backhand) for an additional 30° degrees. Reflux of blood resulted in varying degrees of hyphema (none of which needed postoperative intervention). The blood and viscoelastic were irrigated from the AC and the AC was filled with an air bubble (approximately 50%). Some surgeons advocate that the eye should be pressurized to 15 mmHg or higher after surgery to reduce the risk of hyphema. The corneal wounds were hydrated at the end of the surgery and checked for water-tight closure. The degree of treated trabecular meshwork was directly evaluated during surgery (i.e., 90° up to 120°).

After surgery, patients were treated with prednisolone acetate 1% eye drops (Infanefran forte, Allergan, Frankfurt am Main, Germany) every two hours for one week, followed by three times daily for two weeks postoperatively to avoid infection and inflammation. Additionally, some experts recommend that the patient is asked to maintain a seated position (45–90°) as much as possible over the next 4 days after surgery to minimize blood reflux and hyphema.

### Statistical analysis

Data were analyzed using MATLAB (Version R2022a for Windows) as well as Graph Pad Prism (Version 9.0 for Windows). Normally distributed data were analyzed using the Fisher exact test, whereas non-normally distributed data were compared using the Mann–Whitney and Wilcoxon test. Furthermore, Repeated Measures ANOVA with Tukey’s Multiple Comparison Test was used for further Analysis.

## Results

Overall 69 eyes of glaucoma patients were included in this study. 55 suffered from POAG, and 14 patients were classified as NTG. All patients were using IOP lowering medication prior to the surgery and showed visually significant cataract.

**Fig. 1:**
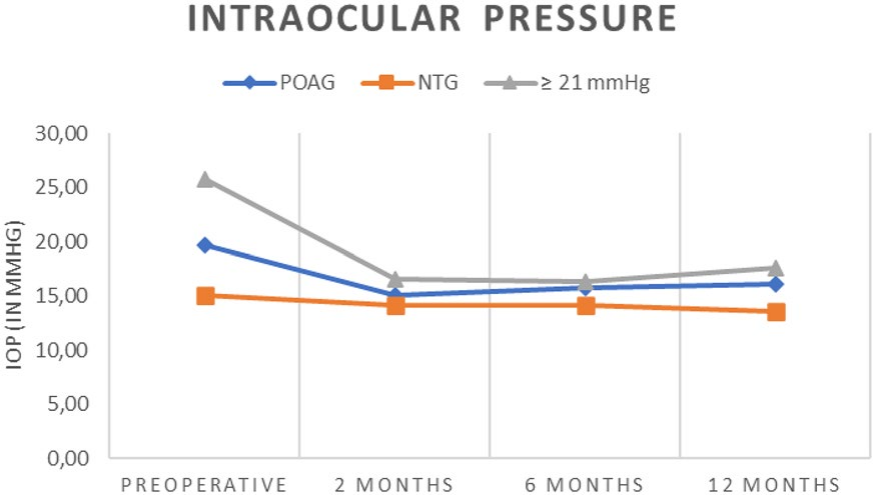
Showing the intraocular pressure (IOP) over the course of the observation period for primary open-angle glaucoma (POAG) patients,  normal tension glaucoma (NTG) patients as well as patients with preoperative IOP ≥ 21 mmHg

Intraocular pressure data at baseline as well as each follow up point are given in Table 1 and Fig. 1.

**Table 1:** is showing the intraocular pressure (IOP) values as well as number of drugs taken by patients suffering from primary open-angle glaucoma (POAG) and normal tension glaucoma (NTG) prior to and 6 and 12 months after surgery

		Preoperative	2 months postoperative	6 months postoperative	12 months postoperative
POAG (n = 55)	IOP (in mmHg) ± SD	19.7 ± 4.5	15.1 ± 2.7	15.8 ± 2.3	16.1 ± 3.2
	IOP lowering medication (number of drugs)	1.7 ± 0.9	0.5 ± 0.8	0.5 ± 0.8	0.6 ± 0.8
NTG (n = 14)	IOP (in mmHg) ± SD	15.1 ± 2.5	14.1 ± 2.4	14.1 ± 3.1	13.6 ± 1.8
	IOP lowering medication (number of drugs)	1.2 ± 0.4	0.3 ± 0.5	0.3 ± 0.5	0.3 ± 0.5

*POAG* Primary open angle glaucoma, *NTG* normal tension glaucoma, *IOP* intraocular pressure, *SD* standard deviation

**Fig. 2:**
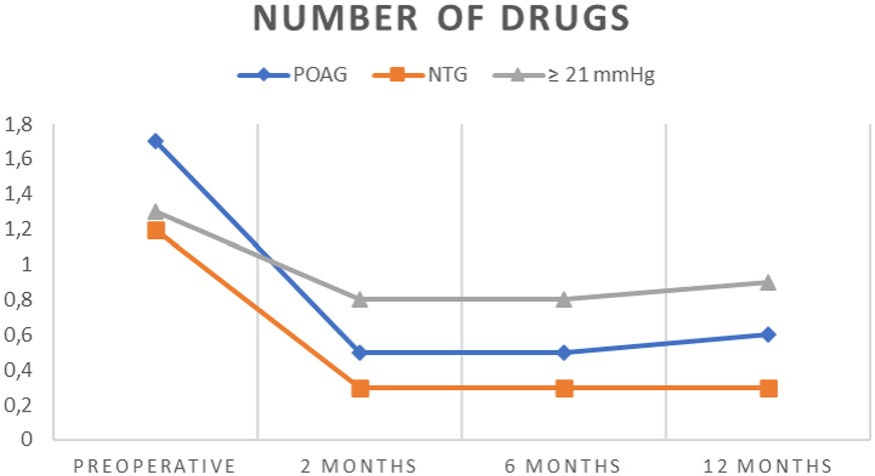
Showing the number of drugs taken over the course of the observation period for primary open-angle glaucoma (POAG) patients, normal tension glaucoma (NTG) patients as well as patients with preoperative IOP ≥ 21 mmHg

In POAG, from a mean preoperative IOP of 19.7 ± 4.5 taking 1.7 ± 0.9 IOP lowering medication, the postoperative IOP reduction was significant at each observation visit (*p *< 0.0001 Repeated Measures ANOVA with Tukey’s Multiple Comparison Test) ranging from 4.6 ± 5.4 at 2 months, 3.9 ± 5.5 at 6 months and 3.6 ± 5.3 mmHg at 12 months. This corresponds to a 19.2% mean IOP reduction at 2 months, 15.3% at 6 months and 14.6% at 12 months. Using the Tukey’s Multiple Comparison Test no significant differences were recorded between the IOPs at each visit after surgery. IOP lowering medication was concordantly reduced by 1.2 ± 1.2 at 2 months, 1.2 ± 1.2 at 6 months and 1.1 ± 1.2 at 12 months (*p *< 0.0001 Repeated Measures ANOVA with Tukey’s Multiple Comparison Test). Resulting in a reduction in the mean number of drugs taken by 68.2% at 2 months, 71.6% at 6 months and 64.5% at 12 months. No significant differences in the number of drugs taken after surgery were recorded using the Tukey’s Multiple Comparison Test. Please refer to Table 1 and Fig. 2

Complete surgical success defined as IOP < 21 mmHg without medication was achieved in 64% of the patients at 12 months.

The proportion of eyes achieving at least 20% IOP reduction from baseline ranged from 54.5% at 2 months and 32.7% at 6 months to 32.7% at 12 months. The proportion of patients with at least 1 IOP lowering medication reduction was 76.4% at 2 months, 81.8% at 6 months and 74.5% at 12 months.

For the 14 NTG patients, preoperative mean IOP was 15.1 ± 2.5 mmHg with 1.3 ± 0.5 anti-glaucomatous medication. The IOP reduction after surgery failed to reach statistical significance (*p *> 0.08, Repeated Measures ANOVA, with Tukey’s Multiple Comparison Test). Consistently, the percentage of patients with a minimum IOP lowering of 20% to baseline was lower with 21.4% at 2 months, 14.3% at 6 months and 7.1% at 12 months. However, reduction of medication burden was higher (76.4% at each observation point) and reached statistical significance (*p *< 0.0001, Repeated Measures ANOVA). Accordingly, 78.5% of the NTG patients achieved a medication reduction of at least 1 drug throughout the complete observation period, please refer to Fig. 2.

Adjusted complete surgical success defined as IOP < 17 mmHg without medication was achieved in 71% of the 14 patients suffering from NTG at 12 months after surgery.

In patients with IOP ≥ 21 mmHg prior to the surgery (n = 19), complete success was achieved in 53% of these patients. Please refer to Fig. 1 and 2 for further visualization of these patients.

In our sub-analysis, 90-degree goniotomy did not perform significantly different than 120 KDB at 12 months regarding either IOP or IOP lowering medication.

The results for 90° vs 120° Kahook at 12 months are presented in Table 2

**Table 2:** is showing the intraocular pressure (IOP) values as well as number of drugs taken by patients in comparison between 90 degrees of Kahook Dual Blade goniotomy and 120 degrees of Kahook Dual Blade goniotomy

	All cases (69 patients)	90° Kahook (18 patients)	120° Kahook (25 patients)	
IOP (in mmHg) ± SD	15.4 ± 3.1	15.6 ± 2.4	15.0 ± 2.9	*p *> 0.7
IOP lowering medication (number of drugs)	0.5 ± 0.8	0.45 ± 0.76	0.6 ± 0.9	*p *> 0.8

*IOP* intraocular pressure, *SD* standard deviation

Mean visual acuity (decimal) improved from 0.58 ± 0.18 to 0.85 ± 0.19 at 12 months (*p *< 0.0001), whereas, visual field defect mean deviation, tested with the standard achromatic protocol (Zeiss Octopus 900, Zeiss, Germany), did not change significantly (− 4.15 ± 5.45 dB and − 4.6 ± 5.78 db, *p *> 0.10).

No sight-threatening complications were encountered in this study. All complications were self-limiting and transient. Please refer to Table 3

**Table 3:** is showing the complications of Phaco- Kahook Dual Blade goniotomy encountered in this study

Complication	Number of patients affected
Self-limiting fibrin reaction	3 (4.3%)
Postoperative macular edema	1 (1.4%)
Postoperative intraocular pressure spike	1 (1.4%)

## Discussion

This prospective case study shows that phacoemulsification combined with Kahook Dual Blade goniotomy can safely and effectively lower IOP in glaucoma patients 12 months after surgery. The surgery is effective in NTG patients as well, whereas IOP reduction did not reach statistical significance. However, complete success for IOP less than 17 mmHg without topical medication was 70% at 1 year. The medication burden was successfully reduced in all POAG and NTG patients.

The overall percental IOP lowering for POAG patients in this study was lower than previously published data. The surgery achieved a 19.2% mean IOP reduction at 2 months, 15.3% at 6 months and 14.6% at 12 months, compared to previously published 26.2% at 12 months after PE + KDB [13]. We believe that this difference is caused by the lower baseline IOP to begin with. As seen in NTG patients IOP was reduced by 10.4% at 2 months, 9.9% at 6 months and 13.3% at 12 months in our study. However, the reduction in medication burden was higher in our study than previously published data (at 12 months: 64.9% vs 50%), this was particularly the case for NTG patients, as medication burden was reduced by 76.4% at 12 months. Furthermore, in our study, more patients were able to reduce their eye drops by at least one agent after 6 months (78.3% versus 63.5%) months after successful Phaco-KDB [14]. Furthermore, the complete success rates of 64% in POAG patients and 71% in NTG patients by a single surgery are promising.

The results of this 1-year follow-up confirm the previously published 6-month results, no significant increase in either IOP or number of topical medication was recorded up to 12 months after surgery (Tukey’s Multiple Comparison Test). However, longer observation periods are necessary to verify these promising preliminary results.

Studies regarding MIGS in NTG patients are quite sparse to date. Neuhann and Neuhann reported a 21.1% IOP reduction at 12 months after PE and I-Stent in 18 NTG patients [15]. Another study by Chang et al. showed a modest IOP reduction and reduction in medication burden in 45 NTG patients. The authors did not publish the different data for the various MIGs procedures and only 5 PE + KDB patients were included in this study [16]. The authors proposed that 2 different MIGS procedures with different mechanisms of actions might achieve better surgical outcomes in NTG patients.

A study of trabeculectomy in 17 NTG eyes in Japan demonstrated an IOP reduction from 13.9 ± 0.9 mmHg to 8.1 ± 2.9 mmHg at 5 years with a decrease in medication burden from 3.0 to 0.8 medications [17]. While this decrease of IOP is significantly higher than that of our study, in part severe complications were observed (transient hypotony in 52.9% (9/17), hypotony maculopathy and VA decline ≥ 0.1 unit in 17.6% (3/17), and hyphema and choroidal detachment in 23.5% (4/17) of eyes) [17]. None of these partially sight-threatening complications were observed in our study. Highlighting the previously proclaimed higher safety rates of MIGS procedures. Although traditional glaucoma filtration surgeries seem to achieve greater long term IOP lowering, MIGS can act as a bridge to such surgeries and spare some patients the possibility of long-lasting surgical complications. Phaco-KDB seems to be a promising technique in glaucoma patients at the time of cataract surgery. The combined surgery is fast, safe and able to achieve significant IOP reduction. However, the technique is neither able to nor aimed at replacing traditional filtrating glaucoma surgeries. In patients with insufficient IOP reduction after Phaco-KDB traditional filtrating surgeries are still feasible. Interestingly, Phaco-KDB was able to significantly lower IOP in patients with decompensated/high IOP prior to the surgery (N = 19) complete surgical success was achieved in > 50% of these patients and none needed further interventions in the first 12 postoperative months. Therefore, Phaco-KDB is a promising technique in phakic glaucoma patients even with decompensated glaucoma and high IOP. At this stage, we would not recommend Phaco-KDB in patients with low target IOP (< 15 mmHg), as the IOP reduction achieved by Phaco-KDB seems to be insufficient in this scenario. Furthermore, Laroche et al. reported that Phaco-KDB was unable to preserve visual field in many patients with advanced glaucoma [18]. Earlier intervention with cataract surgery and MIGS can lower IOP, preserve visual field and reduce spectacle correction [19].

Considering that cataract extraction does significantly lower IOP in glaucoma patients [20], one has to consider, that KDB needs to provide additional IOP reduction/reduction of the medication burden. A recent meta-analysis revealed that phacoemulsification alone is able to reduce IOP by 14% at 12 months and 0.47 for IOP lowering medication [21] and in a study by Majstruk et al. of phacoemulsification in medically controlled POAG, the average preoperative IOP was 17.0 ± 2.7 mmHg with an average reduction of 1.15 mmHg at 1 year, and no significant differences in medication burden were observed at any time [22]. An even smaller IOP-reducing effect of phacoemulsification could potentially be seen in NTG patients, given the lower preoperative IOP in these patients. In a study by Shoji et al., 35 eyes with medically controlled NTG underwent phacoemulsification alone with a statistically not significant IOP decrease from 16.7 to 15.9 mmHg (*p *= 0.195) at 1.5 years and 15.8 mmHg at 2.5 years (*p *= 0.082) with no change in medication burden at either time point [23]. Therefore, it is most likely that the significant reduction in medication burden in our study is the effect of successful KDB.

Our previously published data suggest that traditional success criteria of glaucoma surgeries need to be adjusted for NTG patients. The overall and perecentage reduction in IOP will always be lower in these patients, which might defer surgery. However, the higher reduction in medication burden in these patients seems to be particularly beneficial. Especially, as other factors apart from IOP, particularly intraocular and retrobulbar blood, whereas heatedly discussed [24], might be critical in these patients. The exact effect of topical anti-glaucomatous medication on ocular blood flow is likely due to the nature of these drugs (e.g., beta-blockers), whereas not completely understood today [25].

Finally, our sub-analysis revealed no significant differences between either 90° or 120° degrees of KDB. 90° KDB can often be achieved in one sweeping motion; therefore, it might not be necessary to change the direction of the KDB and perform any of the various developed methods of KDB (e.g., inside-out, outside-in).

### Limitations

The rather small number of patients especially for NTG patients should be taken into consideration when interpreting the results. Nevertheless, to date no other study investigated the success of Phaco-KDB in these patients, making information regarding treatment modalities and surgical success particularly important.

Whereas visual field defect mean deviation did not differ prior to and 12 months after surgery (− 4.15 ± 5.45 dB and − 4.6 ± 5.78 db, *p *> 0.10), this does not complete rule out disease progression in our patients. Longer observation periods as well as further parameters (e.g., optic nerve structural analysis) are necessary. Especially, as one might expect the visual field testing results to improve with better visual acuity after surgery.

Furthermore, as all surgeries were performed by the same surgeon in one center, the results might differ in other surgery centers and other surgeons.

## Conclusion

Combined Phaco-KDB is able to safely and successfully lower IOP in glaucoma patients with significant cataract. In our study, particularly the medication burden was significantly lowered in NTG patients. These results were higher than previously published studies. No significant differences were recorded in terms of 90° vs 120° KDB; therefore, the extend and technique of KDB are up to the users ‘ discretion. Combined Phaco-KDB is a promising novel technique that can be used in POAG patients successfully. In NTG patients up to 70% of the patients may reach sufficient IOP control without the need for topical IOP lowering treatment after one year.
